# Bilateral Perinephric Pseudocysts in an Owl Monkey

**DOI:** 10.1111/jmp.70035

**Published:** 2025-10-06

**Authors:** Alfonso S. Gozalo, Lynn E. Lambert, William R. Elkins

**Affiliations:** ^1^ Comparative Medicine Branch National Institute of Allergy and Infectious Diseases, National Institutes of Health Bethesda Maryland USA; ^2^ Laboratory of Malaria Immunology and Vaccinology National Institute of Allergy and Infectious Diseases, National Institutes of Health Bethesda Maryland USA

**Keywords:** Aotus, capsular cyst, capsular hydronephrosis, capsulogenic renal cyst, nonhuman primate, paranephric pseudocyst, pararenal pseudocyst, perirenal pseudocyst, Primates

## Abstract

Perinephric pseudocysts consist of variable accumulations of either urine, lymph, or blood in a fibrous sac surrounding one or both kidneys. Perinephric pseudocysts are occasionally reported in cats and humans and very rarely in other species. Here we describe a case of bilateral perinephric pseudocysts in an owl monkey.

## 1 Introduction

Perinephric (Perirenal) pseudocysts consist of variable accumulations of fluid in a fibrous sac surrounding one or both kidneys [1]. The term pseudocyst is used to describe this lesion because the cyst is not lined by epithelium [1]. Perinephric pseudocysts are uncommon lesions in cats and humans, and even rarer in other species [1, 2, 3, 4, 5, 6, 7, 8, 9, 10, 11, 12]. Here we describe a case of bilateral perinephric pseudocysts in an owl monkey.

## 2 Case Report

**FIGURE 1:**
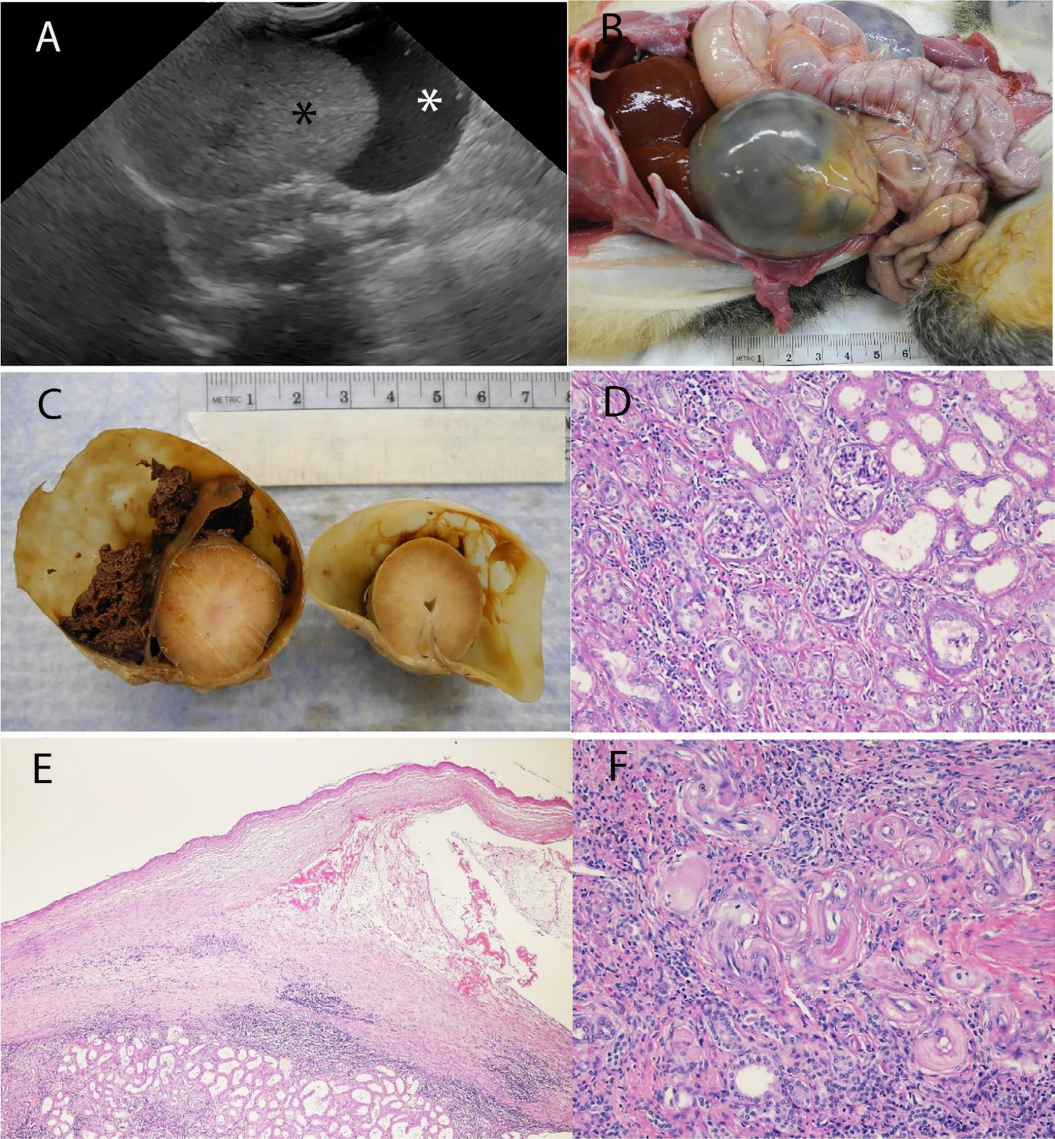
(A) *Aotus nancymai*. Ultrasound image of the right kidney long axis with a perinephric pseudocyst. The kidney is partially surrounded by anechoic fluid (white asterisk). The wall of the perinephric pseudocyst is not clearly visible. The kidney appears normal size but with an increase in the echogenicity of the medulla resulting in a reduction of normal corticomedullary distinction (black asterisk). (B) *Aotus nancymai*. Postmortem image showing the enlarged kidneys. (C) *Aotus nancymai*. Cross section of the kidneys post fixation showing markedly dilated renal capsules. The left kidney contains blood in the subcapsular space. (D) *Aotus nancymai*. Kidney. Image showing renal interstitial fibrosis with nonsuppurative inflammation, glomerular sclerosis, and dilated renal tubules. HE, 200×. (E) *Aotus nancymai*. Kidney. Low power image showing severe inflammation of the renal cortex with separation of the capsule. HE, 100×. (F) *Aotus nancymai*. Kidney. Image showing on cross section several blood vessels with marked hyaline arteriopathy and hyperplastic arteriosclerosis. HE, 200×.

A 3‐year and 2‐month‐old, 1 kg, research naïve, captive‐born, male, owl monkey ( *Aotus nancymai* ) was enrolled in an Institutional Animal Care and Use Committee‐approved research study at the National Institute of Allergy and Infectious Diseases (NIAID), National Institutes of Health (NIH). The monkey was housed and cared for in accordance with the Animal Welfare Act [13], Animal Welfare Regulations [14], and the Guide for the Care and Use of Laboratory Animals [15] in an AAALAC‐accredited animal facility. Housing conditions have been described in detail before [16]. The animal had a history of diarrhea that was treated and resolved, and one instance of a suspected syncopal episode; however, electrocardiographic and chest x‐ray examinations were within normal limits. No other significant findings were noted. During the physical examination, two large, smooth, and firm intra‐abdominal masses were palpated that occupied the mid‐abdominal region. The position and shape of the masses suggested bilaterally enlarged kidneys. Ultrasound examination revealed the masses to be bilateral cystic structures associated with the kidneys. The cystic structures were fluid‐filled sacs partially surrounding the kidneys. The left kidney cyst contained anechoic and a small amount of echogenic fluid, and the right kidney contained only anechoic fluid. The wall of the perinephric pseudocysts was not clearly visible. The kidneys within the cystic sacs were considered normal size but with an increase in the echogenicity of the medulla resulting in a reduction of normal corticomedullary distinction (Figure 1A). Based on the ultrasonographic findings, a diagnosis of bilateral perinephric pseudocysts was made. Complete blood count and serum biochemical analysis values were all within normal limits except for neutrophilia (9956/μL) and elevated creatine phosphokinase (1472 IU/L). Semi‐quantitative urinalysis using a urine dipstick test showed the presence of leukocytes (25–75 cells/μL), protein (30 mg/dL), and blood (50 cells/μL). A month later, the clinical condition of the animal deteriorated, showing increased respiratory effort, possibly due to abdominal distension as a result of the enlarged kidneys displacing viscera and compressing the diaphragm. Complete blood count and serum biochemical analysis showed a very low erythrocyte count (2.4 × 10^6^/μL), low hemoglobin (6.2 g/dL), low hematocrit (21%), low MCHC (29%), elevated BUN (39 mg/dL), and elevated creatinine (1.7 mg/dL). Urinalysis was not performed due to the inability to obtain a urine sample. Due to a poor prognosis, the animal was humanely euthanized with a sodium pentobarbital overdose [17]. At necropsy, both kidneys were markedly dilated, measuring the left kidney 8 by 6 by 5 cm and the right kidney 6 by 5 by 4 cm approximately (Figure 1B). The peritoneal cavity had scant serous effusion. All other organs were grossly normal. The kidneys were injected with 10% neutral‐buffered formalin and fixed intact, lower urinary tract, and samples of reproductive tissues and all major organs were also fixed, embedded in paraffin, and processed routinely for Hematoxylin‐Eosin (H&E) stain. Sectioning of the kidneys after fixation showed both kidneys had severe dilation of the subcapsular space with marked distention of the renal capsule. The right kidney had watery fluid and the left kidney had fluid and clotted blood in the space (Figure 1C). Histologically, both kidneys had severe interstitial fibrosis and nonsuppurative inflammation. The renal tubules were dilated with mild multifocal neutrophilic infiltrates, and glomeruli had multifocal sclerosis (Figure 1D,E). Hyaline arteriopathy and hyperplastic arteriosclerosis were found in multiple tissues including the kidney, heart, mesentery, pancreas, and gastrointestinal tract (Figure 1F). The thoracic aorta had intimal fibrosis, and the heart had cardiomegaly and multifocal moderate myocardial fibrosis. Sections of the pancreas had multifocal to coalescing severe loss of acini, prominent small duct proliferation, and interstitial fibrosis with mild mixed inflammatory infiltrates. The liver had marked small bile duct proliferation at portal triads and large numbers of hemosiderophages. The central hepatic veins had perivascular fibrosis. Bone marrow was hypercellular with all hematopoietic cell lines present; erythroid precursors predominated. All other organs were within normal limits.

## 3 Discussion

Perinephric pseudocysts are classified according to their fluid content [1]. Those that contain urine occur due to chronic extravasation of urine between the kidney and the renal capsule, often associated with trauma [4]. Those that contain blood are called perinephric hematoma and are due to mechanical trauma or diseases that cause blood vessel lesions, or defects in coagulation [18, 19]. Those that contain lymph can be caused by mechanical trauma or diseases that affect lymphatics [5]. And those that contain a transudate that cannot be attributed to urine leakage, hemorrhage, or lymph extravasation are considered to have unknown etiology and are usually associated with chronic renal disease [1, 2, 3]. Measuring the creatinine concentration of the fluid in the pseudocyst relative to the serum allows one to distinguish urine from transudate [1]; however, in our case, because the kidneys were injected with fixative in situ, this was not possible. Differential diagnosis for perinephric pseudocysts includes renal cysts found in several heritable, developmental, and acquired diseases, like polycystic kidney disease, juvenile nephronophthisis, acquired renal cystic disease, renal and perirenal abscesses, renal neoplasia (i.e., cystic renal carcinoma), and hydronephrosis [20]. The two most common causes of chronic nephropathy are diabetes and hypertension, followed by primary glomerular disease, autoimmune diseases, lower urinary obstruction, and, less frequently, hereditary renal disease, urinary tract infections, systemic infections, and certain medications [21]. In our case, the monkey showed vascular changes suggestive of arterial hypertension in multiple organs. Severe renal disease in this owl monkey may have resulted in transudation and possibly damage to renal cortex blood vessels, resulting in leakage into the renal subcapsular space.

To our knowledge, perinephric pseudocysts have been reported in seven species, mostly in cats associated with chronic nephropathy, followed by humans associated with renal transplantation, trauma during surgery, or defects in coagulation [1, 2, 4, 5, 6, 18, 19]. Occasional reports of perinephric pseudocysts in other species include two rams; in one case, the origin was undetermined, and in the second case, it was associated with hydronephrosis [7, 8]. In a dog, the condition was associated with severe renal interstitial fibrosis, while in a squirrel monkey, it was found associated with severe glomerulonephropathy and hypertension [9, 10]. In a ferret, it was associated with polycystic kidney disease, and in a C57BL/6J mouse, it was reported secondary to severe hydronephrosis [11, 12]. In our case, the lesions were associated with chronic renal disease, similar to reports in cats, a dog, and a squirrel monkey [1, 2, 9, 10]. Although uncommon, perinephric pseudocysts should be considered in the differential diagnosis of intra‐abdominal masses in owl monkeys.

## Conflicts of Interest

The authors declare no conflicts of interest.

## Data Availability

The data that support the findings of this study are available from the corresponding author upon reasonable request.
